# European journalists and the sea: Contexts, motivations, and difficulties

**DOI:** 10.1177/09636625221137036

**Published:** 2022-11-30

**Authors:** Bruno Pinto, Ana Matias

**Affiliations:** MARE - Marine and Environmental Sciences Centre / ARNET - Aquatic Research Network, Faculdade de Ciências da Universidade de Lisboa, Portugal; CIMA - Centro de Investigação Marinha e Ambiental / ARNET - Aquatic Research Network, Universidade do Algarve, Portugal

**Keywords:** European newspapers, marine issues, qualitative analysis, science journalism

## Abstract

The media play an important role in informing us about new developments in our understanding of the sea and raising awareness about its sustainability. However, press coverage of marine issues seems to be modest, compared with the importance oceans have in our lives. In this study, we examine science journalists’ working contexts, motivations, and difficulties in writing about the sea in Europe. We conducted semi-structured interviews with 26 journalists who write for quality newspapers from 13 European countries. We found that the recent production of press news on marine issues is mainly conditioned by working contexts in newspapers, the personal and professional interests of journalists, and the available resources to write news. More studies are needed to compare our findings, including with other regions outside Europe.

## 1. Introduction

Human activities are known to be threatening the integrity of coastal and marine environments. Examples of human-induced effects include overfishing, habitat destruction, impacts of climate change, the introduction of exotic species, and coastal erosion (e.g. Boonstra et al., 2015; Duarte et al., 2015). However, many pressing threats are not well known by citizens, which can represent a significant barrier to positive social change (e.g. Kopke et al., 2019).

Research efforts of the past decades on media coverage of marine issues suggest a low presence of these themes (e.g. Pinto et al., 2020; Thompson-Saud et al., 2018). For instance, research on a newspaper in Chile found about 10 news stories per month between 2011 and 2013, representing about 0.6% of news in the newspaper (Thompson-Saud et al., 2018). The main theme was national aquaculture, an important economic activity in Chile. In the Portuguese quality press, there was a similar number of news stories per month between October 2002 and December 2010. In this case, the focus was often marine pollution, marine species and habitats, and fisheries (Pinto et al., 2020). In another study based on four major US newspapers, the search for news stories about marine science published between July 2001 and February 2015 resulted in a fairly low number of 169 news pieces (Johns and Jacquet, 2018). Also, studies have shown that European audiences consider pollution and overfishing as two areas of higher public concern (EU, 2017, 2021; Gelcich et al., 2014; Potts et al., 2016).

Another of the issues that permeate the past 15 years of research concerning science journalism in Europe and North America is the decline of quality newspapers and the professional conditions of journalists (e.g. Bauer and Gregory, 2007; Dunwoody, 2014; Massarani et al., 2021). A global survey of journalists from these two regions indicated a degradation in the traditional business model for news, mainly due to a reduction in the readership and the transition to Internet-based platforms (Bauer et al., 2013). In a follow-up of this study, the proportion of science journalists in Europe/Russia who believed they would certainly work in science journalism in the next 5 years decreased from 34% in 2013 to 28% in 2021 (Massarani et al., 2021). Although these studies are not strictly comparable, these and other indicators suggest a downward pattern in the satisfaction level of journalists.

Considering the discrepancy between the importance of oceans and the modest frequency of news about the sea suggested in the literature, we talked with European journalists to better understand their working contexts, motivations, and difficulties in writing about marine issues. These include themes such as climate change, pollution, biodiversity, fisheries, management, ocean properties, or economic activities. We found that the quality production of press news concerning these issues is mainly conditioned by the working contexts in newspapers, the personal and professional interests of journalists, and the available resources to write news. These results are discussed in the last section, as are the main limitations of this study.

## 2. Methods

**Figure 1.**
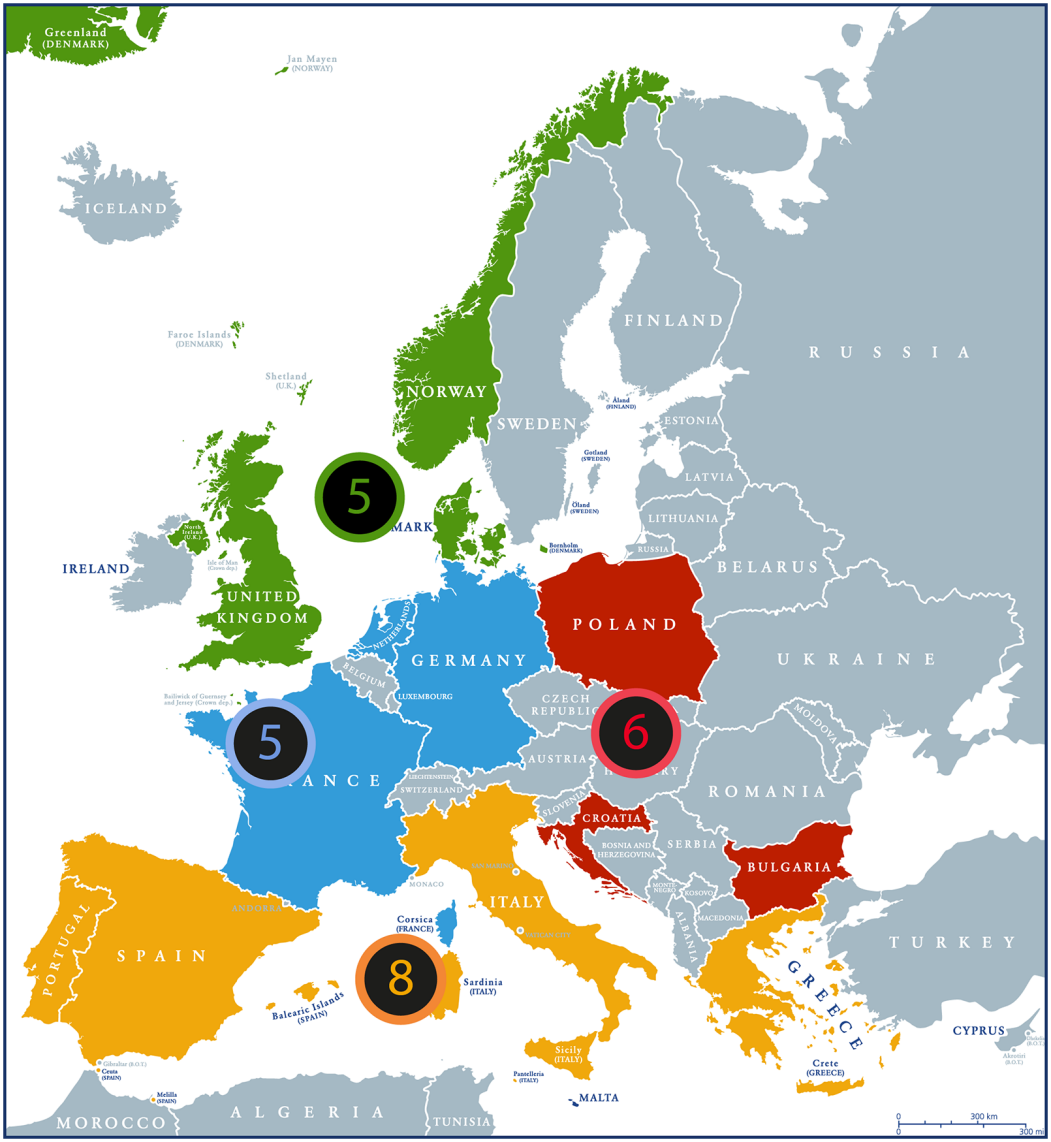
Map representing the countries and number of journalists interviewed in each of the four European regions considered in this study (Northern in green, Southern in yellow, Western in blue and Eastern in red).

Data was collected through 26 semi-structured interviews with science and environment journalists from 13 European countries between February and May 2021. During this period, Europe and the rest of the World were facing the COVID-19 pandemic. In selecting countries, we aimed to cover four different regions of Europe: Northern, Southern, Western, and Eastern (Figure 1). We decided to choose journalists who published in quality newspapers in paper and digital formats in each of the selected countries since these usually set the national news agenda (Boykoff, 2009; Nisbet and Lewenstein, 2002). In the current research, quality newspapers were defined as aiming to provide comprehensive coverage and analysis of international and national news of the day together with informed comments on economic, political, and social issues (adapted from Entwistle and Hancock-Beaulieu, 1992). National quality newspapers for each country were analyzed to identify journalists who had published recently (past 5 years) about the oceans. Respondents were recruited through direct contact with journalists and editors, and indirectly through newspaper contacts and journalist associations.

The main objectives of the interviews were to gather information about the working context of journalists, motivations, and difficulties in writing news about marine issues. Questions asked included the following: What are the greatest difficulties in writing news about marine issues? What motivates you to write about these issues? At the beginning of your career as a journalist, what was your learning/training in writing about science and environment? The full description of the protocol is available at Pinto and Matias (2022). Interviewees were asked to consider their activities beyond the period of the COVID-19 pandemic.

All participants provided written informed consent for participation. Interviews were done remotely using an Internet video call program, lasting between 20 and 60 minutes. Both authors were involved in conducting the interviews, which were digitally recorded, and later transcribed in full, and anonymized. After reading all the interviews, the two authors defined the key themes.

The obtained data were coded using the software NVivo (version 1.5.1; QSR International) and then grouped and analyzed qualitatively according to the defined list of key themes (as described in e.g. Braun & Clarke, 2012; Bryman, 2012). In some cases, it was necessary to add themes that were not in the original list. Coding was also done to organize the interviews’ content regarding motivations for and difficulties in reporting about the sea, although there were sometimes issues in coding and code merging due to the range of responses. Codes related to the context were identified during content analysis, mostly concerning newspaper organization, the relationship of the country to the sea, and national media situations. Additionally, we added quantitative information to the qualitative analysis when this was considered relevant (e.g. Sandelowski et al., 2009).

During cross-check analysis, there were differences in the classification between the two authors, which were resolved through discussion until reaching a consensus. In some cases, this resulted in changing or merging codes.

## 3. Results

Interviewees’ age spanned 25 and 64 years, with an average of about 47 years; 14 were male and 12 female. Almost half of the journalists (*n* = 12, 46%) had initial academic training in science, environment, and technology, with 10 mentioning an undergraduate or postgraduate qualification in journalism. It was decided to include journalists at different stages of their careers, including both journal staff (*n* = 18, 69%) and freelance professionals (Table 1). Four senior interviewees worked both as journalists and editors of the science and/or environmental sections.

**Table 1.** Characterization of interviewed journalists.

Number of journalists interviewed		26
Region (country)	Northern Europe (England, Denmark, Norway)	5 (1,2,2)
	Southern Europe (Portugal, Italy, Greece, Spain)	8 (2,3,2,1)
	Western Europe (France, Germany, The Netherlands)	7 (2,4,1)
	Eastern Europe (Croatia, Bulgaria, Poland)	6 (3,1,2)
Gender	Male	14
	Female	12
Age	Average	47
	Maximum	64
	Minimum	25
Training*	No degree	1
	Journalism degree	10
	Science, Environment & Technology degree	12
	Social sciences & humanities degree	7
Career status	Newspaper staff	18
	Freelancer	8

* Some journalists have graduations in more than one area.

### Working contexts: Three different levels

Interviewees’ descriptions of their working context pointed to three levels of resources for marine science and environmental journalism (see Table 2): the first level concerns more than sufficient resources for these activities, the second level concerns countries with sufficient resources, and the third level concerns countries with insufficient resources to cover these issues.

**Table 2.** Identified levels of marine science and environmental journalism in Europe.

		Level 1	Level 2	Level 3
Journal	Typical team size (science and environment)	6–12	3–5	1–2
	Countries	England France Germany Spain The Netherlands	Denmark Italy Norway Portugal Poland	Bulgaria Croatia Greece
News	Science and environment	Every day	Frequent	Irregular
	Marine	Regular	Moderate—not regular*	Moderate—low coverage*
	Diversity of marine themes	High	Moderate (on environment) Low (on science)	Low

* Depends on internal/external factors such as national agenda, international events, economic dependence on the sea, and cultural and political context.

Research on newspaper circulation was also done to interpret results (sources, e.g. Alliance pour les Chiffres de la Presse et des Médias (ACPM), 2022, for France; Associação Portuguesa para o Controlo de Tiragem e Circulação (APCT), 2022 for Portugal; Audit Bureau of Circulation (ABC), 2022, for the United Kingdom), although we were unable to access updated and reliable numbers of printed copies per day for all newspapers.

The first level includes countries such as Germany and France, in which the sections dedicated to science and the environment are well established in the newspapers, have a regular presence in the press, and have bigger and more diversified teams that cover a wide range of themes. According to numbers reported by several interviewees, the main newspapers of these countries typically have 6–12 science and environmental journalists, and print more than 200,000 copies per day (e.g. in France Le Monde or Le Figaro; source: ACPM, 2022).

The second level includes countries like Italy or Denmark, in which science and environmental issues are present in the newspapers, but the news is written by smaller teams and journalists accumulate different science and environment issues. In these cases, there is a less regular presence of such news in the newspapers. Journalists we interviewed indicated that science and environment newsrooms on national newspapers from this—sufficient—level usually have three to five journalists.

The third level includes countries such as Greece and Croatia, where science and environmental journalists expressed opinions about the lack of specialized staff in these fields and the generally low quality of coverage. At this level, it was mentioned that the relevance of science and environment news still needs to be negotiated, thus such news may be absent in some daily prints. For example, it was said that

[. . .] it’s not very easy because I have to persuade my boss to give me space for that [news on the environment]. (J26)

The number of science and environment journalists at this level is one to two per newspaper, who in some cases may need to cover other topics. Considering the quality of news in this third level, one of the journalists said,

You have coverage of science in the media [. . .], but besides some exceptions, it’s more like copy and paste stuff [from international news]. [. . .] I think it would be good if the mainstream media started to realize that they need more specialized people. (J1)

Interviewees also reported different levels of national interests related to marine issues, which seems to be higher in countries with large coasts and/or islands. In the words of a journalist from Greece,

In general, we are almost a country of islands. And even if you live on the mainland, you are very connected to the sea. [. . .] So, there is a lot of interest in these issues. (J20)

In contrast, journalists from Poland and Germany mentioned they perceived an overall detachment of audiences concerning the sea. A journalist from Germany said,

[. . .] the forest would be much more important in our national mindset than the sea. (J14)

Twelve interviewees said that science reporting since early 2020 has concentrated on the COVID-19 pandemic, which also increased their usual workload. At the same time, some journalists stated this has been important to highlight the work of science journalists and contributed to an increase in the presence of science in the media.

Nevertheless, journalists working in all three contexts—insufficient, sufficient, or more than sufficient levels—almost all agreed that there is not enough attention to marine issues. A journalist and editor of a Level 1 journal expressed his or her opinion as follows:

[. . .] I mean, it’s just frustrating with so many people, and still it’s just not enough. It’s not enough. It’s dramatic. At the moment, we have nobody just for climate change. (J6)

This comment is reminiscent of journalists working in a level three context, except the amount/variety of news and desk size are different.

### Motivations: Personal interest, professional duties, and making a difference

Almost all the interviewees (*n* = 25) had one or more degrees from different backgrounds (see Table 1 for details), but often learning about journalism on the job was deemed very important (even the most important). For instance,

I don’t think studying journalism is a good way. To be a journalist it’s [. . .] a profession like [. . .] shoemaker, so we learn it through practice [. . .]. (J8)

The most frequently mentioned motivation for journalists to write about science and environmental themes was personal interest. Some interviewees (*n* = 10) talked about how they are personally connected to nature and/or the sea. For example,

[. . .] the main thing for me to write about these themes altogether [environmental issues], is that I’m very engaged [. . .] It’s very important for me [. . .] So that drives me [. . .]. (J23)

Also, several journalists (*n* = 7) mentioned their experiences growing up and/or spending free time by the sea and even finding hobbies related to the marine environment. For instance,

I started writing about environmental issues and that led me to become interested in diving. So, first I was a journalist and then I became a diver. (J21)

Moreover, interviewees perceived themes such as climate change and marine pollution as highly important and mentioned their interest in books, documentaries, news, websites, and other media about the sea, and the desire to contribute to the protection of habitats, species, or specific areas. Indeed, in several interviews (*n* = 6), a sense of mission and of working to make a difference was noticed. For instance, environmental issues were linked to people’s health and welfare. A journalist stated,

[. . .] people are not very concerned by a threat [climate change] that is so far away in time. Because we have a very short-term view. (J4)

Interviewees also talked more broadly about the challenges of understanding and communicating complex issues (*n* = 5), as well as the possibility of interacting with scientists and other protagonists (*n* = 7). There were references to professional duties such as reporting on pollution accidents, published scientific studies or events such as the annual conferences, and published reports of the Intergovernmental Panel on Climate Change (IPCC). Occasionally, it was mentioned that requests from the editors, professional recognition, and higher income from freelance jobs were drivers for journalists to write about the sea.

### Difficulties: Lack of information and resources

When asked about difficulties, several interviewees (*n* = 9) talked about getting information from the right scientists, and governmental organizations, and accessing appropriate data to tell a story. For example,

So, the Ministry of Environment in the last few years [. . .] is very non-cooperative when it comes to journalists’ questions. [. . .] (J3)

They also mentioned the struggles of choosing appropriate themes and dealing with the complexity of some issues. Counting on the help of a specialist or, in bigger newspapers, resorting to freelance journalists who specialize in certain topics were both mentioned as ways to address this challenge.

Interviewees also talked about internal difficulties within the newsrooms, usually referring to a lack of resources such as time, money, and opportunities to report outside the office. It was curious to notice that even journalists from the first level of journalism context (identified before) talked about the difficulties of ensuring good media coverage of science and the environment due to the lack of time and specialized journalists. Also, four interviewees said that these issues were not a priority for their newspaper, which relates to the context of journalism previously mentioned.

## 4. Discussion

In this article, we identified three main factors that condition the activities of European journalists who write news about marine issues: their working contexts, their personal and professional motivations, and resources available to produce news. When a journalist works in a big newsroom, with more than sufficient resources, it is possible to publish regular news about the sea and to cover a wider range of themes. Journalists and editors can work on marine topics because there is enough staff and time to cover different scientific and environmental areas of interest and, in some cases, the possibility to hire freelance journalists. We also found that, in these cases, even in countries where marine issues are not part of the dominant cultural setting and the journalist does not express a particular interest in these themes, news of marine-related science and environment is written as part of wide overall science coverage. However, it should be noted that journalists working in these newspapers think that more should be done to report scientific advances and raise awareness of marine problems, particularly those related to climate change.

However, different working contexts alone do not explain the frequency of news about marine issues in contexts with fewer resources. In these cases, results showed that personal interest, professional duty, or a perceived national relation to marine issues influences the production of news, including topics that are not so relevant on the agenda (e.g. reporting aboard scientific vessels). This personal interest and connection were shown in interviews in a variety of forms, including growing up near the sea, summer holidays at the beach, hobbies such as scuba diving, and past career occupations such as biologist or navy merchant. Other researchers had noticed that a passion for science can be a major motivation for science journalists (Ashwell, 2016; Bauer et al., 2013). Our results reinforce this, which was also associated with the wish to contribute to a societal challenge. This was expressed in terms of the importance of the sea for human life and sustainability and is especially visible in issues such as climate change, biodiversity, or fisheries. Additionally, there was a sense of professional duty when reporting on issues considered important, for example, international events or publications. This confirms the results of a previous study which found an equilibrium in the activities of science journalists in three different countries between their interests and a set of common news factors such as immediacy and interest to target audiences (Rosen et al., 2016).

The main difficulties in writing news about the sea, were scarce resources and the struggle to access information. Similarly to the previous findings, interviewees usually talked about their scarce time, money, and opportunities to report outside the office, and some mentioned low editorial priority attributed to science and the environment as limitations to the writing of news (as noted by Davies et al., 2021; Massarani et al., 2021 and Schäfer, 2017). However, we did not identify the perception of an ongoing shift in the working conditions of interviewees recently reported by other authors (e.g. Davies et al., 2021). Instead, we found certain stability and resistance to pressures, as mentioned by Ashwell (2016) in New Zealand. The explanation for this probably lies in the characteristics of our sample—most interviewees were staff journalists in national quality newspapers—with additional and valued work due to the news coverage of the pandemic.

One of the main limitations of the current research was the fact that, in some cases, only one journalist per country was interviewed (e.g. Spain, England, the Netherlands, and Bulgaria). Although it was considered that these interviews provided an overview of the press coverage in these countries, information about national contexts such as differences between newspapers within the same country was probably less detailed. Moreover, there is little research on the activities of science and environmental journalists in Europe and even less on marine issues. With scant information from previous studies about journalists’ main difficulties and motivations, we were less able to make comparisons between the past and present. Since we interviewed only European journalists, future research about these issues could explore other regions of the world. Additionally, the assessment of perspectives of newspaper editors and directors in future research would help to better understand editorial decisions related to the news coverage of marine issues. Furthermore, since data collection was done during the second global COVID-19 lockdown, it seems possible that the conditions of participants for interviews varied (due to factors such as workload, homeschooling, etc.).

Despite these limitations, results showed consistent patterns across countries that the recent production of news on marine issues in Europe was mainly conditioned by available resources in newspapers and the personal and professional interests of journalists. Based on our findings, apart from the personal and professional interest of the journalists, all other identified factors are external: national context, newspaper context, and editorial choices, which implies that change is complex. Therefore, the possibility of a higher presence of marine news in the future seems to be dependent on the external pressure of society on the media, for example, from readers, politicians, scientists, youngsters, activists, and nongovernmental organizations (NGOs). Considering the importance that the sea represents to our societies, the threats to its sustainability, and its direct connection to climate change, these findings should be taken into consideration when planning for marine science and environmental communication.
